# The causes of maternal mortality in adolescents in low and middle income countries: a systematic review of the literature

**DOI:** 10.1186/s12884-016-1120-8

**Published:** 2016-11-11

**Authors:** Sarah Neal, Shanti Mahendra, Krishna Bose, Alma Virginia Camacho, Matthews Mathai, Andrea Nove, Felipe Santana, Zoë Matthews

**Affiliations:** 1 Department of Social Statistics and Demography, University of Southampton, Southampton, UK; 2 Options Consultancy Services, London, UK; 3 Sustainable Development and Health, Ferney Voltaire, France; 4 SRH Team, Latin America and the Caribbean Regional Office, United Nations Population Fund, Panama City, Panama; 5 Centre for Maternal and Newborn Health, Liverpool School of Tropical Medicine , Liverpool, UK; 6 Novametrics, Derby, United Kingdom; 7 Instituto Nacional de Endocrinología, Havana, Cuba

## Abstract

**Background:**

While the main causes of maternal mortality in low and middle income countries are well understood, less is known about whether patterns for causes of maternal deaths among adolescents are the same as for older women. This study systematically reviews the literature on cause of maternal death in adolescence. Where possible we compare the main causes for adolescents with those for older women to ascertain differences and similarity in mortality patterns.

**Methods:**

An initial search for papers and grey literature in English, Spanish and Portuguese was carried out using a number of electronic databases based on a pre-determined search strategy. The outcome of interest was the proportion of maternal deaths amongst adolescents by cause of death. A total of 15 papers met the inclusion criteria established in the study protocol.

**Results:**

The main causes of maternal mortality in adolescents are similar to those of older women: hypertensive disorders, haemorrhage, abortion and sepsis. However some studies indicated country or regional differences in the relative magnitudes of specific causes of adolescent maternal mortality. When compared with causes of death for older women, hypertensive disorders were found to be a more important cause of mortality for adolescents in a number of studies in a range of settings. In terms of indirect causes of death, there are indications that malaria is a particularly important cause of adolescent maternal mortality in some countries.

**Conclusion:**

The main causes of maternal mortality in adolescents are broadly similar to those for older women, although the findings suggest some heterogeneity between countries and regions. However there is evidence that the relative importance of specific causes may differ for this younger age group compared to women over the age of 20 years. In particular hypertensive conditions make up a larger share of maternal deaths in adolescents than older women. Further, large scale studies are needed to investigate this question further.

## Background

While adolescent fertility has declined steadily over the last few decades, every year an estimated 16 million adolescents still give birth between the ages of 15 and 19 [1], and as many as one million become mothers before the age of 15 years [2]. The potential health risks as well as social and economic disadvantage faced by these young women and their infants is widely recognised, as evidenced by the inclusion of adolescent fertility as a key indicator for reproductive health in the recently concluded Millennium Development Goals [3].

While recent studies suggest the increased risk of maternal mortality faced by adolescents is not as great as previously believed [4, 5], pregnancy-related conditions are still a major cause of death among adolescent girls and young women, with an estimated 15 % of all deaths globally in women aged 10–25 years being a result of maternal causes [6]. In addition children of adolescent mothers are more likely to experience adverse outcomes such as perinatal or neonatal death, and their infants are more likely to be born prematurely or have low birth weight (e.g. [7, 8]). There is some evidence that these disadvantages are particularly concentrated among younger adolescents [7], but others suggest the risk persists through to older adolescence [9].

The main causes of maternal mortality for women of all ages are well documented. A recent systematic review [10] suggests that around three quarters of all maternal deaths globally are a result of direct obstetric causes: haemorrhage is the leading global cause of maternal death (27 % of all maternal deaths) followed by hypertensive disorders (14 %) and sepsis (10 %). Other important direct causes are abortion (8 %) and embolism (3 %). While the main causes of maternal death for women of all ages were similar across all regions, the study found significant regional variation for the proportion each cause contributes to total maternal mortality. Around 27 % of maternal deaths for all ages are from indirect causes, but less is known about the specific conditions that contribute to this figure [11].

Much less is known about whether causes of mortality are different for adolescent girls than for older women: adolescents have important physiological differences due to their relative physical immaturity, as well as demographic and socio-economic characteristics that may place them at greater risk from particular causes of death. This systematic review aims to ascertain the main causes of maternal death in young women under the age of 20 years, and examine whether they differ from causes of maternal death in older women.

## Methods

Our study was designed to comply with the Preferred Reporting Items for Systematic Reviews and Meta-Analyses (PRISMA) statement [12], and in order to make our study replicable, we developed and followed a clearly defined protocol. We initially searched the following datasets for papers in English, Spanish and Portuguese published since 1974 to February 2015: Pubmed (which includes Medline), Embase, CINAHL, POPLINE, the Latin American and Caribbean Health Sciences Literature database (LILACS), and the Index Medicus for the World Health Organization’s Africa, South East Asia and Eastern Mediterranean regions (there were no comparable resources for other WHO regions). We also searched the Cochrane library and the WHO Reproductive Health Library. A summary of search terms used (which were adapted somewhat for different databases) is included in the Appendix. Other types of literature (grey) were sought through search engines such as Google and through websites of relevant organisations. We also searched the references of relevant articles for further papers.

Only studies from low and middle income countries (as defined by the World Bank [13]) were included. No particular methodologies were specified in the inclusion criteria, and we included both published and unpublished studies where relevant. The outcome of interest was the proportion of adolescent maternal deaths due to each cause, and therefore studies presenting their findings as comparative risks were excluded. “Adolescent” was defined as below the age of 20, but no lower age limit was set. Studies with fewer than 10 deaths among those aged under 20 years were excluded as it was not deemed that these could provide useful information on the distribution of causes.

One team (SM and SN) carried out the review of the English language papers, and a separate team (AVC and FS) reviewed the Spanish and Portuguese literature. One review author from each team performed the search and the initial screening of the titles and abstracts. Studies judged to be potentially eligible for inclusion and studies of uncertain but possible relevance were retrieved in full. These were then reviewed by two authors independently to apply the eligibility criteria outlined above. Any disagreement was resolved by discussion. Data extracted were entered into a predesigned data extraction form based on the criteria proposed by the Strengthening the Reporting of Observational Studies in Epidemiology (STROBE) statement [14] and the Transparent Reporting of Evaluations with Non-randomized Designs (TREND) statement [15]. The key information included: country/region, study design/methodology, sampling, data source, number of deaths reported (including the number of adolescent maternal deaths), description of the population studied and the study setting (context) and results – i.e. cause of death. Where available, we also extracted data on cause of death in older age groups for comparative purposes.

Maternal death was defined as “the death of a woman while pregnant or within 42 days of termination of pregnancy, irrespective of the duration and site of the pregnancy, from any cause related to or aggravated by the pregnancy or its management but not from accidental or incidental causes^1^” [16]. If papers included incidental causes these were removed, and the proportions recalculated. Two papers contained numbers of cases for each cause of maternal death, along with an estimate of numbers for all maternal deaths in the sample but did not report the data in a form which fulfilled our criteria i.e. mortality from all causes [17] or proportion in each age group classed as having died from each cause [18] was presented. In these cases proportions were recalculated. One paper [19] included age- and cause-specific maternal mortality ratio (MMR), along with number of births by age group. Numbers were derived from the MMR, and the proportions calculated using these numbers.

The authors made an assessment of the quality of each paper and risk of bias based on a checklist tool which drew on existing literature on the evaluation of cross-sectional and observational studies [20, 21]. Criteria included: sample size, description of the study population and sampling criteria, methods of analysis, data collection tools and methods, and level of missing data. The studies were rated as low, medium or high quality by two assessors, and any differences were managed by discussion.

Studies of causes of maternal mortality overall suggest geographical variation [10], so findings were grouped by region. We used WHO regions for this, slightly adapted by the use of “sub-Saharan Africa”, rather than “Africa”, because no studies from North African countries were identified during the review. Because the groupings of causes were not consistent across the studies, and we expected heterogeneity between regions, meta-analysis was not appropriate so the results are written up and presented as a narrative synthesis.

**Fig. 1:**
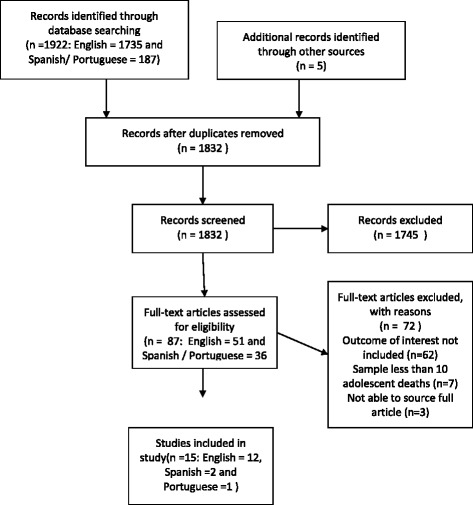
PRISMA flow diagram

The search of the databases yielded 1735 English language abstracts and 187 Spanish and Portuguese language abstracts. Following their assessment 51 English and 36 Spanish and Portuguese full texts were requested of which three were unobtainable (see Fig. 1 for PRISMA Flow Chart). Of the final 15 papers included (12 in English, two in Spanish and one in Portuguese), nine were from sub-Saharan Africa, two from the South East Asia region and four from the Pan American Health Organisation (PAHO) region (see Table 1 for full details of the papers). Of the 69 full text papers examined and not included in the final group, 62 were excluded as they did not contain the outcome of interest as defined in the protocol (proportion of adolescent maternal deaths due to each cause), and the remaining seven were excluded as there were fewer than 10 deaths in the study. Two studies used the same data [18, 22] but both were included as they presented slightly different findings and results. Seven studies were based on retrospective analysis of institutional records, two were prospective hospital-based studies, one was based on official national statistics, three studies (including two based on the same data source) used retrospective community-based survey data, one was based on community surveillance data, and one used both official statistics and hospital records.

Based on the criteria outlined in the methodology section, five papers were judged to be moderate quality, one of high quality with the rest being judged as low quality. The main problems identified were small sample size, lack of clarity or limited explanation of data collection or classification methods, lack of description of study population, lack of representativeness of sample (in particular many studies were based in tertiary facilities) and lack of measures of variability. More details can be found on these limitations in the discussion.

## Results

A summary of the findings from each paper can be found in the evidence grid in Table 1. The majority of the adolescent maternal deaths were from direct causes (between 54 % and 88 % in studies that distinguished between direct and indirect causes) i.e. resulting from a complication of pregnancy, delivery, or management of these events. In particular, the most commonly cited causes were haemorrhage, hypertensive disorders, sepsis, abortion and obstructed labour. See Table 2 for a summary of findings by main direct causes.

**Table 1:** Evidence grid based on included papers

Study	Site	Study design/data collection methods	Years	Adolescent age group	Number of adolescent deaths included	Causes of maternal deaths identified		
Sub-Saharan Africa							Adolescent group	Comparison group (where available)
Asamoah B, Moussa K, Stafstrom M, Musinguzi G (2011) [18]	Ghana	Retrospective community survey with verbal autopsy questionnaire (Demographic and Household Survey)	2000–2005	12–19 years (as part of sample 12–49 years: 12–14 and 15–19 were presented separately but were amalgamated when figures recalculated due to only three deaths in 12–14 age group)	65 (as a sub- sample of 605 maternal deaths aged 12–49)		12–19 years	12–49 years
						*Total Direct*	*62 %*	*61 %*
						Abortion	29 %	14 %
						Haemorrhage	11 %	23 %
						(Pre) eclampsia	8 %	N/A
						Sepsis	6 %	7 %
						Obstructed labour	5 %	5 %
						Miscarriage	3 %	3 %
						Hypertensive disorders	N/A	9 %
						*Total* * Indirect*	*38 %*	*40 %*
						Other infectious diseases	14 %	14 %
						Other non-infectious diseases	12 %	12 %
						Miscellaneous	12 %	14 %
						Figures were recalculated because the data were presented as cause specific mortality by age group rather than adolescent mortality by cause		
Ghana Statistical Service (GSS), Ghana Health Service (GHS), and Macro International (2009) [22]	Ghana	Retrospective community survey with verbal autopsy questionnaire (Demographic and Household Survey)	2000–2005	12–14 and 15–19 (as part of sample 12–49 years: 12–14 and 15–19 were presented separately but were amalgamated when figures recalculated due to only three deaths in 12–14 age group)	56 aged 12–19 (as a sub-sample of 486 maternal deaths aged 12–49)		**12–19 years**	**12–49 years**
						Abortion	29 %	11 %
						Haemorrhage	12 %	24 %
						Hypertensive disorders	9 %	9 %
						Sepsis	7 %	7 %
						Miscarriage	4 %	4 %
						Obstructed labour	3 %	4 %
						Other miscellaneous	9 %	13 %
						Other not classified elsewhere: infectious	15 %	15 %
						Other not classified elsewhere: non-infectious	12 %	13 %
Mallé D, Ross DA, Campbell O, Huttly S (1994) [23]	Mali (Bamako, Sissako and Mopti)	Retrospective study using data from 25 health facilities	1988 to 1992	14–19 years	54 aged 14–19 as a sub-sample of 288 maternal deaths aged 14–49		**14–19 years**	**14–49 years**
						Haemorrhage	46 %	59 %
						Toxaemia	30 %	14 %
						Infection	17 %	13 %
						Other	7 %	14 %
Granja A CL, Machungo F, et al. (2001) [24]	Mozambique	Retrospective hospital based Study	1989–1993	Under 20 (with a comparison non-adolescent group aged 20–45)	52 (with a further 80 non-adolescent deaths)		**<20 years**	**20–45 years**
						*Total Direct*	*54 %*	*61 %*
						Pregnancy-induced hypertension/eclampsia	21 %	9 %*
						Puerperal infection	15 %	10 %
						Abortion	10 %	7 %
						Haemorrhage	4 %	33 %**
						Amniotic fluid embolism	4 %	2 %
						*Total Indirect*	*47 %*	*38 %*
						Malaria	27 %	12 %**
						Anaemia	6 %	6 %
						Meningitis	6 %	4 %
						Other indirect	6 %	16 %
Lema VM, Changole J, Kanyighe C, Malunga EV (2005) [29]	Malawi	Retrospective study using tertiary health facility data	1999–2000	10§§–19 years (as part of a sample aged 16–40)	42 (as a subset of the total sample of 204 maternal deaths aged 10–40)		**10–19 years**	**16–40 years**
						Postabortal sepsis	36 %	N/A
						Puerperal sepsis	21 %	29 %
						Postabortal haemorrhage	10 %	N/A
						Ectopic pregnancy	10 %	3 %
						Meningitis	10 %	10 %
						Pneumonia	10 %	N/A
						Eclampsia	2 %	N/A
						Other	2 %	6 %
						Postabortal complications	N/A	N/A
						Other infections	N/A	24 %
						Obstetric haemorrhage	N/A	20 %
						Ruptured uterus/obstructed labour	N/A	11 %
						Heart failure (due to anaemia)	N/A	3 %
						Not established	N/A	2 %
								3 %
Nkata M (1997) [28]	Zambia (Luapula province)	Retrospective study using secondary level hospital data	1993–5	13–19 years	33		**13–19 years**	**No comparison data available**
						*Total Direct*	*69 %*	
						Sepsis due to:		
						- Obstructed labour with uterine rupture	36 %	
						- Obstructed labour without uterine rupture	9 %	
						- Abortion	9 %	
						- Puerperal sepsis	3 %	
						Haemorrhage due to:		
						- Caesarean section	6 %	
						- Ectopic pregnancy	3 %	
						Eclampsia	3 %	
						*Total Indirect*	*30 %*	
						Malaria	18 %	
						HIV infection	6 %	
						Meningitis	3 %	
Airede LR and Ekele BA (2003) [27]	Nigeria	Retrospective hospital based Study	1990–1999	<19 years	46		**<19 years**	**No comparison data available**
						Eclampsia	46 %	
						Prolonged obstructed labour	30 %	
						Anaemia	11 %	
						Ruptured uterus	7 %	
						Postpartum haemorrhage	4 %	
						Abortion	4 %	
						Puerperal sepsis	4 %	
						Meningitis	2 %	
						Congestive cardiac failure	2 %	
						Sickle cell disease	2 %	
						Total is over 100 % as in some cases two independent causes were included)		
Ujah I, Aisien O, Muthihir J, et al. (2005) [25]	Nigeria (Plateau State)	Prospective study using tertiary health facility data	1991–2001	10–19 years	25		**10–19 years**	**No comparison data available**
						*Total Direct*	*76 %*	
						Abortion	28 %	
						Sepsis	20 %	
						Eclampsia	20 %	
						Haemorrhage	8 %	
						*Total Indirect*	*24 %*	
						Sickle cell anaemia	8 %	
						Other indirect causes	16 %	
Okusanya B, Algere E, Abe A, Ibrahim H and Salawu R (2013) [26]	Nigeria (Katsina State)	Prospective study using maternal mortality database in tertiary health facility	2008–2012	15–19 years	16		** 15–19 years**	**15–45 years**
						Pre-eclampsia/eclampsia	37.5 %	18.9 %
						Obstructed labour/uterine rupture	13 %	6.8 %
						Puerperal sepsis	13 %	12.2 %
						Tetanus in puerperium	13 %	5.4 %
						Haemorrhage	12 %	21.6
						Sickle cell anaemia	6 %	N/A
						Anaemia	6 %	8.1
South East Asia								
Fauveau V, Koenig MA, Chakraborty J, et al. (1988) [30]	Bangladesh (Matlab)	Data gathered from community based demographic surveillance system, with additional information gathered from health staff, health records and interviews with families	1976–1985	15-19 years	78 adolescent deaths (originally 94 but 16 deaths from incidental causes removed and results recalculated) as a subsample of 387 deaths age 15–45 years		**15–19 years**	**20–34 years**
						*Total Direct*	*88 %*	*85 %*
						Toxaemia/eclampsia	22 %	14 %
						Abortion	20 %	16 %
						Postpartum haemorrhage	18 %	21 %
						Other obstetric	14 %	18 %
						Obstructed labour	7 %	7 %
						Postpartum sepsis	6 %	8 %
						*Concomitant*	*12 %*	*14 %*
						Medical causes	7 %	7 %
						Unspecified	5 %	7 %
Yusuf HR, Akhter HH, Chowdhury ME, Rochat RW (2007) [17]	Bangladesh	Data and health care provider interviews from 4751 health facilities in Bangladesh.	1995/6	10–19 years	10–19 1038 as a sub-sample of 8821 maternal deaths aged 10–50		**10–19 years**	**10–50 years**
						*Total Direct*	*89 %*	*88 %*
						Eclampsia	47 %	29 %
						Abortion-related causes	12 %	18 %
						PPH / APH	10 %	10 %
						Obstructed labour	9 %	14 %
						Retained placenta	5 %	8 %
						Other obstructed causes	4 %	7 %
						Tetanus	2 %	2 %
						*Total Indirect*	*11 %*	*12 %*
Latin America and Caribbean/PAHO								
Souza ML, Burgardt D, Ferreira LAP, Bub MBC, Monticelli M, Lentz HE (2010) [31]	Santa Catarina, Brazil	Retrospective population based study	1994–2005	10–19 years	58 (originally 64, but 6 deaths classified as “unrelated” were removed and proportions recalculated)		**10–19 years**	**No comparison data available**
						*Total Direct*	*74 %*	
						Toxaemia	29 %	
						Infection	17 %	
						Haemorrhage	16 %	
						Other direct	12 %	
						*Total Indirect*	*26 %*	
de Siqueira AAF, Tanaka ACd'A (1986) [32]	Brazil	Retrospective review of health records and official statistics	1980	10–19 years (10–14 and 15–19 years were presented separately but were amalgamated for this study due to small number of deaths in 12–14 age group)	306 (18 aged 10–14 288 aged 15–19 years)		**10–19 years**	**No comparison data available**
						Hypertensive disorders	47 %	
						Sepsis	16 %	
						Haemorrhage	8 %	
						Abortion	6 %	
						Embolism	2 %	
						Other pregnancy-related causes	12 %	
						Other	7 %	
Donoso ES and Carvajal C (2012) [19]	Chile	Retrospective study of statistics from the yearbooks of Chile’s National Institute of Statistics	2000–2009	15–19 years	36 as a sub-sample of 431 maternal deaths aged 15–49		**15–19 years**	**20–49 years**
						Hypertensive disorders	44 %	30 %
						Abortion	18 %	11 %
						Obstetric embolism	6 %	6 %
						Postpartum haemorrhage	3 %	6 %
						Puerperal sepsis	3 %	5 %
						Ectopic pregnancy	N/A	7 %
						Concurrent illness	28 %	35 %
						Percentages were calculated based on births within each age groups and numbers of deaths by cause within each age group.		
Acosta Chavez (2003) [33]	Peru	Retrospective from tertiary hospital	1986–2002	Under 20 years	30		**<20 years**	**No comparison data available**
						*Total Direct*	*70 %*	
						Septic abortion	33 %	
						Infections	17 %	
						Haemorrhage	13 %	
						Toxaemia	7 %	
						*Total Indirect*	*30 %*	
						Pulmonary tuberculosis	10 %	
						Liver failure	7 %	
						Others	13 %	

§These figures were not presented in the paper, but were provided by the author in a personal communication

§§There is a discrepancy in the paper on youngest age

*/**difference between adolescents and non-adolescents significant at 5 %/1 % level

**Table 2:** Range of estimates of % maternal deaths in adolescents attributable to five main direct causes

	Range of estimates from included studies of % maternal deaths in adolescents attributable to cause
Haemorrhage	3 % (Donoso 2012) [19] 4 % (Airede et al. 2003) [27] 4 % (Granja et al. 2001) [24] 8 % (Ujah et al. 2005) [25] 8 % (de Siqueira 1986) [32] 9 % (Nkata 1997) [28] 10 % (Lema et al. 2005) *Post-abortal haemorrhage only* 10 % (Yusuf et al. 2007) [17] 11 % (Asamoah et al. 2011) [18] 12 % (Ghana Statistical Services et al. 2009) [22] 12 % (Okusanya et al. 2013) [26] 13 % (Acosta Chavez 2003) [33] 16 % (Souza et al. 2010) [31] 18 % (Fauveau et al. 1988) [30] 46 % (Mallé et al. 1994) [23]
Hypertensive disorder	2 % (Lema et al. 2005) 3 % (Nkata 1997) [28] 7 % (Acosta Chavez 2003) [33] 8 % (Asamoah et al. 2011) [18] 9 % (Ghana Statistical Services et al. 2009) [22] 20 % (Ujah et al. 2005) [25] 21 % (Granja et al. 2001) [24] 22 % (Fauveau et al. 1988) [30] 29 % (Souza et al. 2010) [31] 30 % (Mallé et al. 1994) [23] 38 % (Okusanya et al. 2013) [26] 44 % (Donoso 2012) [19] 46 % (Airede et al. 2003) [27] 47 % (Yusuf et al. 2007) [17] 47 % (de Siqueira 1986) [32]
Maternal peripartum sepsis	3 % (Donoso 2012) [19] 4 % (Airede et al. 2003) [27] 6 % (Asamoah et al. 2011) [18] 6 % (Fauveau et al. 1988) [30] 7 % (Ghana Statistical Services et al. 2009) [22] 13 % (Okusanya et al. 2013) [26] 15 % (Granja et al. 2001) [24] 16 % (de Siqueira 1986) [32] 17 % (Mallé et al. 1994) *Classified as “infection”* [23] 17 % (Souza et al. 2010) *Classified as “infection”* [31] 17 % (Acosta Chavez 2003) [33] 20 % (Ujah et al. 2005) [25] 21 % (Lema et al. 2005) *Not including post-abortal sepsis* 48 % (Nkata 1997) *Includes sepsis resulting from obstructed labour* [28]
Obstructed labour	3 % (Ghana Statistical Services et al. 2009) [22] 5 % (Asamoah et al. 2011) [18] 7 % (Fauveau et al. 1988) [30] 9 % (Yusuf et al. 2007) [17] 13 % (Okusanya et al. 2013) [26] 30 % (Airede et al. 2003) [27] 45 % (Nkata 1997) *Includes sepsis resulting from obstructed labour* [28]
Abortion	4 % (Airede et al. 2003) [27] 6 % (de Siqueira 1986) [32] 9 % (Nkata 1997) [28] 10 % (Granja et al. 2001) [24] 12 % (Yusuf et al. 2007) [17] 3 % (Donoso 2012) [19] 20 % (Fauveau et al. 1988) [30] 28 % (Ujah et al. 2005) [25] 29 % (Asamoah et al. 2011) [18] 29 % (Ghana Statistical Services et al. 2009) [22] 33 % (Acosta Chavez 2003) [33] 36 % (Lema et al. 2005) *Post-abortal haemorrhage and infection*

### Haemorrhage

Maternal haemorrhage is excessive bleeding from the genital tract and is the main cause of maternal death globally. It can occur during pregnancy (antepartum), during childbirth (intrapartum) or after childbirth (postpartum), but in general the studies did not differentiate. Overall, the systematic review found that although haemorrhage contributed to a fair proportion of maternal deaths amongst adolescents, it was not the leading cause of death amongst them. If we examine the sub-Saharan African countries, in Mali [23] 46 % of the 54 adolescent maternal deaths were as a result of haemorrhage. However, most of the other studies found markedly lower percentages of deaths assigned to this cause: in Ghana 11 and 12 % of deaths [18, 22] and in Mozambique [24] 4 % of deaths in adolescents were attributed to this cause. The three Nigerian studies found that deaths due to haemorrhage contributed 8 % [25] 12 % [26] and 4 % [27] respectively to the total adolescent maternal deaths. The remaining two sub-Saharan African studies are somewhat more difficult to interpret: Nkata et al. [28] attributed 9 % of deaths to haemorrhage in the Zambian study, but these were specifically linked to ectopic pregnancy and caesarean section. In Malawi, Lema et al. [29] attributed 10 % of deaths to post-abortal haemorrhage, but gave no figure for haemorrhage with other underlying causes. Most of these estimates are low compared to the estimate of 25 % for Sub Saharan African reported by Say et al. [10]. There is also some evidence that the proportion of deaths due to haemorrhage is lower amongst adolescents than in older women. Even in the Mallé et al.’s study [23], where nearly 50 % of adolescent maternal deaths in Mali were due to haemorrhage; this is lower than the estimate for older women (60 % for 20–35 year olds and 71 % for women over 35 years).

Two studies in Bangladesh estimated that 10 % [17] and 18 % [30] of the adolescent maternal deaths were due to haemorrhage. Again this is markedly lower than the 30 % assigned by Say et al. [10] but figures for older women are broadly comparable across these studies. In Latin America, Souza et al.’s study [31] found that 16 % of adolescent deaths in Brazil were caused by haemorrhage, whereas de Sequeira et al.’s (1986) study, also in Brazil [32], which had a larger sample size estimated this to be 8 %. Studies in Peru [33] and Chile [19] attributed 13 and 8 % respectively to this cause. Again, this is markedly lower than the figure assigned by Say et al. [10] for this region.

### Hypertensive disorders, pre-eclampsia and eclampsia

Pre–eclampsia is defined as hypertension in pregnancy with onset after 20 weeks gestation with proteinurea, whereas eclampsia is defined as convulsions in a woman with signs of pre-eclampsia [34]. Hypertensive disorders (which include eclampsia and pre-eclampsia as well as the pre-existing hypertension) are amongst the most common morbidities to occur during pregnancy. Hypertensive disorders were also found to be a major cause of death in a number of studies for the adolescent age group, and the data suggest it may make up a greater percentage of deaths among this group than for the older ages.

The three Nigerian studies found that 46 % [27], 20 % [25] and 38 % [26] of adolescent maternal deaths could be attributed to eclampsia. Okusanya et al. [26] provides a comparable estimate of 19 % for the entire sample aged 15–45 years. In Mali [23] 30 % of adolescents died of pre-eclampsia as opposed to 12 % in women aged 14–49 years and in Mozambique [24] eclampsia caused 21 % of the adolescent maternal deaths: a significantly greater proportion than for older women (9 %). These proportions were seen to be low in only two studies: a study in Zambia found that 3 % of adolescent deaths were attributable to eclampsia [28] and in Malawi only 2 % were due to this cause [29]. These figures are generally markedly higher than the 16 % suggested for eclampsia in sub-Saharan Africa by Say et al. [10].

The two Bangladeshi studies found that 22 % [30] and 47 % [17] of adolescent maternal deaths were attributable to pre-eclampsia or eclampsia. These estimates were markedly higher in adolescents than for older age groups in both studies. With the exception of Acosta Chavez’s study [33] in Peru, hypertensive disorders were the leading cause of death for adolescents across the various studies from Latin America, ranging from 29 % [31] to 47 % [32], and these are markedly higher than comparative figures (22 %) given by Say et al. [10] for Latin America.

### Maternal peripartum sepsis

Peripartum or puerperal sepsis can be defined as a bacterial infection of the genital tract occurring between the onset of rupture of membranes or labour and the 42nd day postpartum [35]. Our review shows this to be another important cause of death among adolescents, but estimates varied widely. In Zambia Nkata [28] found over 50 % of adolescent maternal deaths were due to sepsis, but these included sepsis resulting from obstructed labour (with or without uterine rupture) which might be categorised differently in other studies. The three Nigerian studies found that 20 % [26], 13 % [27] and 4 % [28] of adolescent maternal deaths were a result of sepsis. Research in Mali [23] found 17 % of all adolescent maternal deaths were from this cause, with similar findings of 15 % in Mozambique [24]. An estimate of 21 % was presented from the Malawian study [29] but were lower in the Ghanaian studies [18, 22]. In Bangladesh sepsis was not recorded as a cause of any maternal deaths in Yusuf et al.’s study [17], but made up 6 % of adolescent maternal deaths in Fauveau et al.’s paper [30]. Where estimates were also available from other age groups, no discernible patterns were evident: whilst in some studies the estimates for adolescents were somewhat higher [23, 24], in others they were lower [29] and in some they were very similar [18, 26, 30]. Three of the Latin American studies had very similar estimates of 16 or 17 %, whereas the fourth was much lower at 3 % [19].

### Obstructed labour and ruptured uterus

Labour is classed as obstructed when the presenting part of the fetus cannot progress into the pelvis despite strong contractions [36]. Evidence from the studies was somewhat confusing on this, partly because of the way the cause of death was defined (e.g. the causes were variously categorised as obstructed labour, ruptured uterus, sepsis resulting from obstructed labour/ruptured uterus), and a number of studies did not report this cause at all [19, 23–25, 29, 31–33].

One Nigerian study suggests 30 % of maternal deaths were due to “prolonged obstructed labour” [27] with a further 7 % due to ruptured uterus, while another [26] suggests a much lower estimate of 13 %. The Zambian study shows 45 % of adolescent maternal deaths were due to sepsis caused as a result of obstructed labour with or without uterine rupture [28]. Asamoah et al. [18] show that 5 % of adolescent maternal deaths result from this cause in Ghana, and the Ghana Statistical Service (GSS) estimates this at 3 % [22]. In Bangladesh, Yusuf et al. [17] and Fauveau et al. [30] found that 9 and 7 % of deaths respectively were due to obstructed labour (with Yusuf assigning a further 4 % to “other obstructed causes”). Only four studies provide comparisons with older age groups: in three [18, 22, 30] the proportion of deaths assigned to this cause is the same or very similar, but in one [23] it is higher in adolescents. However, most estimates were larger than Say et al.’s [10] estimates for sub Saharan Africa and Southern Asia, which were less than 3 %. None of the studies from Latin America provided specific data on obstructed labour.

### Abortion-related death

The term abortion refers to expulsion of the products of conception from the uterus before the fetus is viable [37], and can be either spontaneous or induced. Induced abortion can be either ‘safe’ or ‘unsafe’ based on how it is conducted. An abortion is considered ‘unsafe’ if the procedure for terminating an unwanted pregnancy is conducted either by persons lacking the necessary skills or in an environment lacking minimal medical standards or both. The overwhelming majority of deaths in such situations result from complications of unsafe abortion.

Few studies, in this review, differentiated the data for abortion by type, and in those that did, the numbers were extremely small for spontaneous abortion so we aggregated them. The data on abortion as a cause of death show very marked differences between the studies. Of the three studies from Nigeria, one reported it to be 4 % [27], while in the second this was 28 % [25], and in the third [26] abortion was not cited as a cause at all. In the Malawian study this was very high, with an estimated 36 % of deaths caused by post-abortal sepsis and a further 10 % from post-abortal haemorrhage [29]. The proportion was also very high in both studies from Ghana [18, 22]. In Mozambique and Zambia the figure was much lower at 10 and 9 % respectively [24, 28]. Figures for the two Bangladeshi studies were 20 % [30] and 12 % [17]. In several studies the proportion of deaths assigned to abortion was markedly higher for adolescents than for older women [18, 29], whereas for others it was similar [24, 30]. Many of the figures are much higher than the 10 % for sub-Saharan Africa and 6 % for Southern Asia suggested by Say et al. [10]. In Latin America estimates were very high in the Mexican study (33 %) [33], and also high in Donoso and Carvajal’s Chilean study at 18 % [19], which is somewhat greater than the 11 % assigned to the total sample group. Both estimates are higher than Say et al.’s [10] estimate of 10 % for Latin America. De Siqueira et al. [33] suggest a lower estimate for Brazil of 6 %.

### Indirect causes

Indirect maternal deaths are those resulting from previously existing diseases, or from diseases developed during pregnancy that were not due to direct obstetric causes but aggravated by the physiological effects of pregnancy.

Where estimates were possible from the data, estimates of the proportion of adolescent maternal deaths due to indirect causes varied widely from 11 % [17] to 47 % [24]. Causes varied by study as well, but for a number of sub-Saharan African studies, infectious diseases were particularly important. Two studies found malaria a particularly important indirect cause of maternal mortality in adolescents. In Mozambique, 27 % of deaths in adolescents were from malaria [24], where it was the highest cause of death, compared to only 12 % in women aged 20 and over. In Zambia malaria was responsible for 18 % of deaths in adolescents [28]. Other indirect causes mentioned included other infectious diseases such as meningitis and tuberculosis, as well as anaemia.

In some countries, human immunodeficiency virus (HIV) infection rates are particularly high among sexually active adolescent girls [38]. However, in the papers included in this review the only study that provides a figure specifically for HIV is from Zambia, which estimated 6 % of adolescent maternal deaths were attributable to this condition [28]. One other study highlighted that it may be difficult to attribute HIV as a cause of death and it may be recorded as another condition [25]. It is also worth noting that some of the studies are very old and data would be from before the HIV epidemic in sub-Saharan Africa became established.

## Discussion

Unsurprisingly, in general the major causes of direct maternal deaths in adolescents are the same as for older women: eclampsia, peripartum sepsis, haemorrhage and abortion. Some studies also show obstructed labour and malaria as important causes of death. However, the contribution of each of these causes differs markedly between studies, which may reflect country or regional differences, although some caution needs to be exercised as it may reflect issues related to definitions or data quality, which are discussed later in this section. In addition some studies show that the main cause or causes of death differ between adolescents and older women.

Because of the limitations of the studies included, it is difficult to draw firm conclusions about how adolescent causes of death may differ from older women. However, there does appear to be reasonable evidence that eclampsia is a more important cause of death in adolescents than in older women. Some studies have confirmed that adolescents are at greater risk of developing pre-eclampia or eclampsia [7] although the physiological pathway is unclear. In addition a very high proportion of adolescent births will be first births, which also carries an increased risk of eclampsia. These findings point to the importance of ensuring adequate antenatal care for the detection and treatment of this condition in adolescents.

Additionally, based on these studies, haemorrhage seems to be a less important cause of death in adolescents than in older women. Aside from eclampsia and haemorrhage, however, findings for most other major causes (e.g. sepsis and abortion) tended to be inconclusive and inconsistent. The data on abortion are extremely heterogeneous and are likely to be prone to a number of limitations and biases that are discussed in the next section. However, several studies indicated unsafe abortion was an important cause of adolescent maternal death. It has been estimated that around 15 % of unsafe abortions are among adolescents [39], but this can vary by context. Many adolescents experience particular barriers in accessing contraception [40, 41], and a focus on providing youth friendly family planning services is therefore an essential component in reducing the burden of adolescent maternal mortality.

It might have been expected that obstructed labour would be a more important cause due to the commonly cited claim that adolescents are at a greater risk of this condition [42]. However, while most estimates from these papers were higher than regional estimates for all age groups by Say et al. [10], we were able to find little comparative evidence from the papers. In addition it is worth noting that obstructed labour may be an important cause of increased morbidity among adolescents, and there is evidence that the risk of fistula is greater among this younger group [43]. Lack of mortality from this condition, particularly in the Latin American studies, may reflect improvements in health services and access to caesarean section.

Some studies suggested very high proportions of deaths from malaria in adolescents, which is concerning. While there is evidence that adolescents are more likely to have malaria infection during pregnancy than older women [44], a study in Uganda highlighted that pregnant adolescent girls tend not to recognise the risks posed by malaria during this time [45]. This again emphasises the need for access to quality antenatal care for adolescents including, where appropriate, intermittent presumptive treatment of malaria.

### Limitations and data quality

Our review is somewhat limited by both the quantity the quality of available studies. Most of the studies have relatively small numbers of adolescent deaths, which obviously makes the analysis of causes problematic due to sampling error. Only one of the studies provided confidence intervals or some other measures of variability for estimates, but we can assume that for most of these studies these would be large, making interpretation difficult. This may partially explain some of the variation, and larger scale studies should be developed in order to address this issue more conclusively. Further potential problems could arise from the method of determining cause of death. The majority of studies obtained cause of death from medical records (often retrospectively) which relies heavily on the skills of available medical staff, and (particularly in cases where the window between admission and death is short) diagnosis may be inaccurate or incomplete. Community based studies used verbal autopsy, which also has been found to provide inconsistent data on cause of maternal deaths [46, 47].

Bias could also be introduced through the fact that the majority of studies were facility based, and mostly took place in large hospitals in major cities. In many countries many births and a proportion of maternal deaths occur at home, so conditions that cause death more rapidly (e.g. haemorrhage) may be under-reported due to many women never reaching a health facility. As adolescent mothers are more likely to be poor and live in rural areas [48, 49], which are also the population groups that are least likely to access health care, or in some contexts may be less likely to seek care [50] this source of bias may differentially affect them. Large scale community based studies overcome this problem, but may carry other potential sources of bias. However, it was not possible to identify any clear differences in major causes between community or hospital based studies in the papers included.

A further problem was lack of consistency between the studies as to how the causes of maternal deaths were categorised: only six of the fifteen studies [17–19, 31–33] attempted to draw on the International Classification of Diseases (ICD) system when grouping causes of death. A recent study [51] demonstrated the feasibility of applying the most recent WHO maternal mortality ICD developed in 2012 [52] to retrospective classification of causes of maternal mortality, and found it a feasible tool for use with existing datasets of maternal death reviews. It has an additional advantage of also allowing underlying and contributing factors to be recorded and analysed separately. Greater consistency among studies would enable more accurate comparisons across studies.

The findings on abortion as a cause of death show great variation not only between countries (which might be expected, particularly in the light of differing laws on obtaining safe abortion) but also within countries where more than one study is available. This is likely to result from a number of causes: in many countries there are marked geographical differences in access to safe abortion services, and ethnic and socio-cultural differences between populations may influence young women’s perceived need to seek an unsafe abortion [53, 54]. However much of the variance is likely to reflect data collection issues or issues of clarification. In the few community-based surveys which rely on information from relatives or community workers, informants may be unwilling to provide the information needed to assign abortion as cause of death. For the hospital-based studies, accuracy and representativeness of data on how the death is classified will depend on whether women are willing or able to attend the hospital when post-abortion complications become evident. There is evidence that abortion deaths are more likely to be classified as “unknown” than other obstetric causes, particularly in contexts where staff may be sanctioned for providing care to women who have sought abortion [55].

This study highlights the paucity of evidence on causes of adolescent maternal death, and emphasises the need for greater efforts to collate and analyse data on age-specific causes of maternal mortality. These data need to be large-scale and based on community surveys using standardised definitions in order to reduce bias and ensure comparability. Data from high-income countries, including those which capture mortality data relating to disadvantaged or marginalised groups, would also be valuable in ascertaining how cause of maternal death among adolescents differs from that among older women.

## Conclusion

The direct causes of adolescent maternal death are broadly similar to those in older women, although their relative importance differs between settings. Furthermore this study provides evidence that the relative importance of the main causes may be different for adolescents than for older women: in particular there is a strong indication that eclampsia and pregnancy induced hypertension may make up a larger proportion of deaths overall than for older women. This demonstrates the needs for all adolescents to access the entire continuum of care during pre-pregnancy, pregnancy, childbirth and the post-partum period. It also emphasises the need for a particular focus on targeted preventative measures aimed at conditions which account for a relatively high proportion of mortality in this group. Further community based studies in a range of countries using large sample sizes and rigorous identification and classification of cause of death are needed to provide more robust evidence on this topic.

## Appendix

**Table 3:** Search Strategy (adapted for different search engines)

A. pregnancy in adolescence
OR
B. (i) Adolescent or teen* or juvenile* or teen* or or minor* or youth or youths or girl or “maternal age” AND (ii) Pregnancy or “pregnant women” or parturition or gestation or maternal or maternity or mother or motherhood or “maternal health services” or “maternal health care” or obstetric or childbirth
AND
C. (i) mortality or death or fatality AND (ii) hemorrhage or sepsis or infection or hypertension or eclampsia or hellp or preeclampsia or obstructed labor or prolonged labor or dystocia or ectopic or embolism or HIV or malaria or tetanus or violence or suicide or abortion

## Abbreviations

HIV: Human immunodeficiency virus
ICD: International Classification of Diseases
LILACS: Latin American and Caribbean Health Sciences Literature database
MMR: Maternal mortality ratio
STROBE: Strengthening the Reporting of Observational Studies in Epidemiology
TREND: Transparent Reporting of Evaluations with Non-randomized Designs
WHO: World Health Organization

## Footnotes

1 Incidental or accidental deaths are those that are not related to the pregnancy e.g. road accident or violence
